# Analysis of the expression of PHTF1 and related genes in acute lymphoblastic leukemia

**DOI:** 10.1186/s12935-015-0242-9

**Published:** 2015-10-05

**Authors:** Xin Huang, Suxia Geng, Jianyu Weng, Zesheng Lu, Lingji Zeng, Minming Li, Chengxin Deng, Xiuli Wu, Yangqiu Li, Xin Du

**Affiliations:** Southern Medical University, 510515 Guangzhou, People’s Republic of China; Department of Haematology, Guangdong General Hospital, Guangdong Academy of Medical Sciences, 510080 Guangzhou, People’s Republic of China; Institute of Hematology, Medical College, Jinan University, 510632 Guangzhou, People’s Republic of China; Key Laboratory for Regenerative Medicine of Ministry of Education, Jinan University, 510632 Guangzhou, People’s Republic of China

**Keywords:** *PHTF1*, *BCL11B*, *FEM1b*, *Apaf*-*1*, ALL, Overexpression, Proliferation, Apoptosis

## Abstract

**Background:**

Previous study showed that downregulated *BCL11B* expression in T cell acute lymphoblastic leukemia (T-ALL) cell line Molt-4 inhibited cell proliferation and induce apoptosis, which may be related to *PHTF1* gene overexpression. The objective of this study was to investigate the expression of *PHTF1* and related genes in ALL and further explore its function in T-ALL cell lines.

**Methods:**

Real-time PCR was used to determine the gene expression level of *PHTF1* in hematologic malignancies. The *PHTF1*, *BCL11B*, *FEM1B* and *Apaf*-*1* gene expression levels and correlations were analyzed in patients with primary ALL (including T-ALL and B-ALL) and healthy individuals (HIs). Inhibition and overexpression of *PHTF1* by lentiviral transduction were performed using the Molt-4 and Jurkat cell lines. Cell growth and apoptosis were measured by the Cell Counting Kit-8 assay and flow cytometry, respectively. Upon *PHTF1* overexpression, the *BCL11B*, *FEM1B* and *Apaf*-*1* gene expression levels were determined by real-time PCR.

**Results:**

*PHTF1* overexpression was found in both T-ALL (p = 0.004) and B-ALL (p < 0.001) groups compared with HIs group. A trend toward a negative correlation between the *PHTF1* and *BCL11B* genes was detected for the T-ALL group, while positively correlated expression was found for the *PHTF1* and *BCL11B* genes in HIs (*P* = 0.001). *FEM1b* and *Apaf*-*1* overexpression was found in recently diagnosed ALL patients compared with HIs (p < 0.05). Positively correlated expression was found for the *PHTF1*, *FEM1b* and *Apaf*-*1* genes in patients with ALL (p < 0.05) and HIs (p < 0.05). Direct up-regulation of *PHTF1* expression inhibited the proliferation of Jurkat and Molt-4 cells and effectively induced apoptosis in Molt-4 cells. Direct inhibition of *PHTF1* expression had no significant effect on the proliferation or apoptosis of Jurkat and Molt-4 cells. *FEM1b* and *Apaf*-*1* overexpression, which did not obviously alter the *BCL11B* expression level, was detected in *PHTF1*-transduced T-ALL cell lines.

**Conclusions:**

*PHTF1* overexpression is responsible for regulating cell proliferation and apoptosis in T-ALL cell lines. *PHTF1* may be a tumor-suppressor like gene and a therapeutic target for triggering the *PHTF1*-*FEM1b*-*Apaf*-*1* apoptosis pathway.

## Background

T cell acute lymphoblastic leukemia (T-ALL) results from clonal malignant T cell proliferation, is an aggressive malignancy that does not respond well to chemotherapy and has a poorer prognosis [1, 2]. The cellular biology and pathogenesis of T-ALL are relatively complex, and these might be related to the different original malignant T cell clone [3–5]. Complex acquired genetic aberrations include chromosomal translocations, gene rearrangements and mutations, resulting in the abnormal expression of oncogenes such as *Notch*1, *TAL1* (T-cell acute lymphoblastic leukemia 1), and *BCL11B* (B-cell chronic lymphocytic leukemia/lymphoma 11B), which may be associated with advanced disease and resistance to treatment [6–10].

The B-cell leukemia/lymphoma 11B (*BCL11B*) gene is a member of the BCL family and plays a crucial role in the development, proliferation, differentiation, and subsequent survival of T cells [11]. *BCL11B* gene alterations are related to the malignant T cell transformation that occurs in hematological malignancies [6, 12–15]. Remarkably, the *BCL11B* gene is responsible for regulating apoptosis and cell proliferation [16–18]. Previous studies [16–18] have shown that inhibition of *BCL11B* expression by siRNA selectively inhibits proliferation and effectively induces apoptosis in T-ALL cell lines (Jurkat and Molt-4) but not in normal mature T and CD34+ cells [17, 19]. Additionally, global gene expression profiling has revealed that *BCL11B* siRNA-mediated apoptosis in Molt-4 cells might be related to the *PHTF1* gene [10].

*PHTF1* (putative homeodomain transcriptional factor) is a putative homeobox gene located at 1p11-p13 in the human genome [20]. This gene is evolutionarily conserved [21] and mainly expressed in the testis [20]. As a transcription factor, the *PHTF1* gene is mainly involved in biological processes such as DNA- dependent transcription and the regulation of biological processes. However, studies on the *PHTF1* gene in leukemia have not been reported. *FEM1b* (feminization-1 homolog b) has been identified as a binding partner for *PHTF1* [22]. Previous in vitro experiments have suggested that human *FEM1b* is involved in apoptosis. *FEM1b* is a proapoptotic protein that interacts with the apoptosis-inducing proteins Fas, tumor necrosis factor receptor-1 (*TNFR1*), and apoptotic protease activating factor-1 (*Apaf*-*1*) [23]. Therefore, we hypothesized that the *BCL11B* gene and the *PHTF1*-*FEM1b*-*Apaf*-*1* pathway may work together in tumor cell apoptosis. In this study, we analyzed the expression level of *PHTF1* and its related genes for the first time in patients with ALL. To further explore its function in T-ALL cell lines, we performed experiments involving the down regulation or overexpression of *PHTF1* in T-ALL cell lines using growth and apoptosis assays in vitro.

## Results

### Expression characteristics and correlation analysis of the *PHTF1* and *BCL11B* genes in T-ALL and B-ALL patients and HIs

**Fig. 1:**
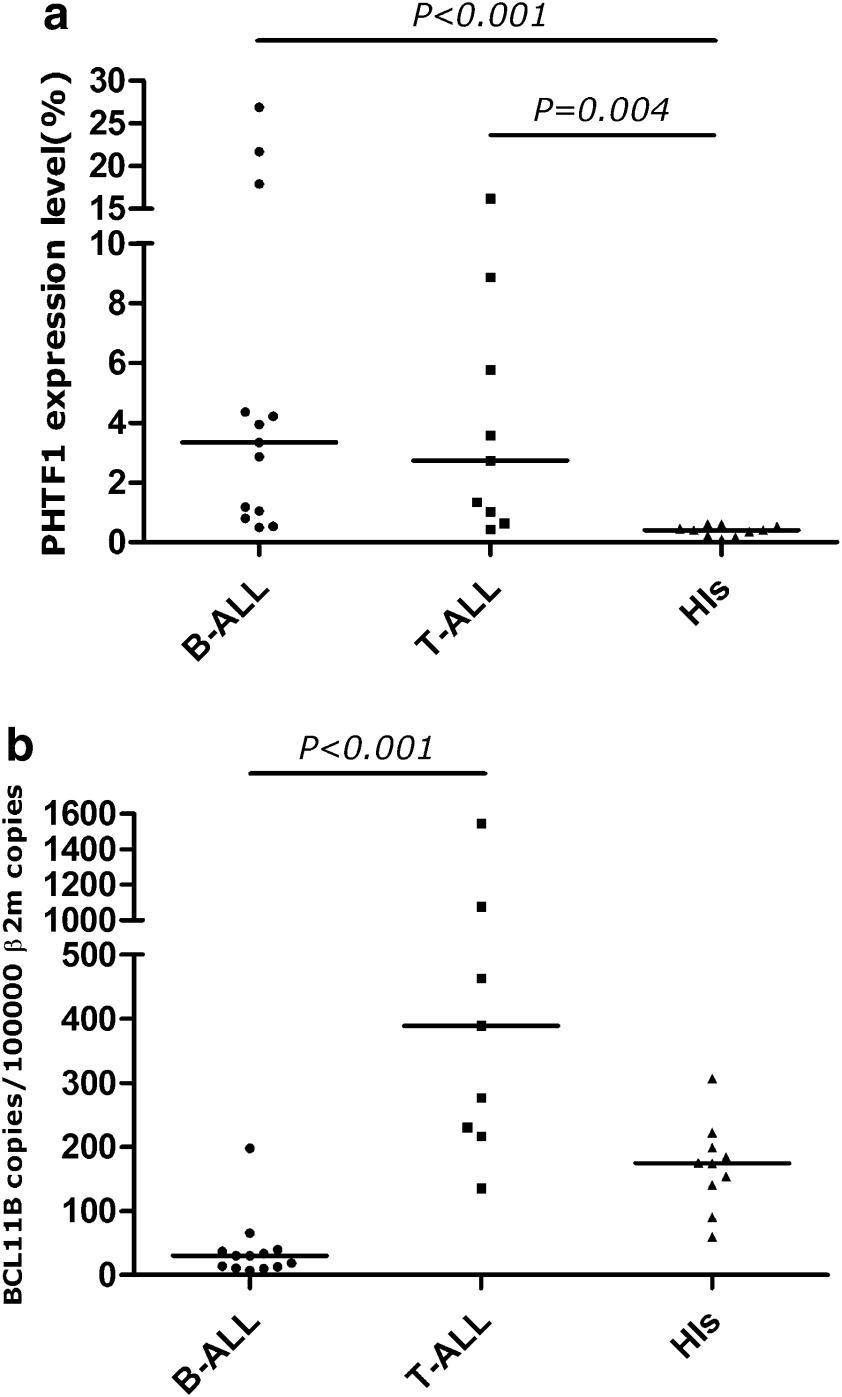
*PHTF1* and *BCL11B* expression level in the different ALL subtypes and healthy individuals. **a** *PHTF1* expression level in the different ALL subtypes and healthy individuals. *PHTF1* overexpression was detected in recently diagnosed T-ALL and B-ALL patients. *P* = 0.004 for T-ALL versus HIs, *P* < 0.001 for B-ALL versus HIs. **b** *BCL11B* expression level in the different ALL subtypes and healthy individuals. *BCL11B* overexpression was detected in patients with T-ALL. *P* < 0.001 for T-ALL versus B-ALL

In order to characterize the expression of *PHTF1* in primary T-ALL samples, we detected the expression level of *PHTF1* in peripheral blood mononuclear cells (PBMCs) from 9 cases with T-ALL (median: 2.73 %, mean rank: 20.33, *P* = 0.004), as well as 13 cases with B-ALL (median: 3.34 %, mean rank: 21.54, *P* < 0.001). Overexpression of *PHTF1* was found in both groups in comparison with HIs group, and there was no significant difference between the two ALL subtypes (Fig. 1a).

As previously reported [24], the *BCL11B* mRNA expression level in PBMCs from patients with T-ALL (median: 389.04 copies/10^5^*β2M* copies, mean rank: 26.56, *P* < 0.001) was significantly higher than that in patients with B-ALL (median: 30.39 copies/10^5^ β2M copies, mean rank: 7.69) (Fig. 1b).

**Fig. 2:**
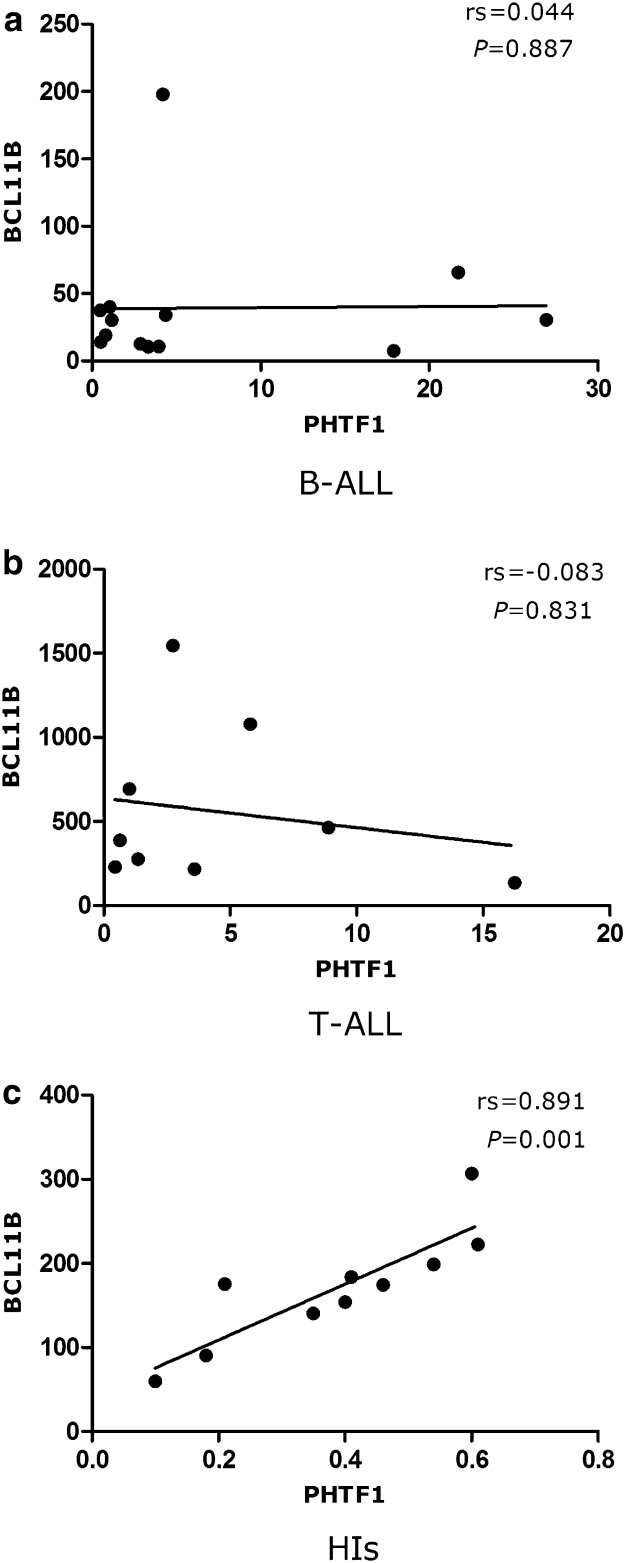
Correlation analyses of the *PHTF1* and *BCL11B* expression levels. **a** No significant correlation was found for the *PHTF1* and *BCL11B* genes in B-ALL patients. **b** A negative correlation trend was detected for the *PHTF1* and *BCL11B* genes in the T-ALL patients. **c** A positively correlated expression level for the PHTF1 and BCL11B genes was found in the HIs

Spearman’s rank correlation analysis of the expression levels of the *PHTF1* and *BCL11B* genes was performed for patients with T-ALL and B-ALL. No significant correlation was found for the *PHTF1* and *BCL11B* genes in the B-ALL patients (rs = 0.044, *P* = 0.887) (Fig. 2a). However, a negative correlation trend was detected for the *PHTF1* and *BCL11B* genes in the T-ALL patient (rs = −0.083, *P* = 0.831) (Fig. 2b). A positively correlated expression level for the *PHTF1* and *BCL11B* genes was found in the HIs (rs = 0.891, *P* = 0.001) (Fig. 2c).

### Overexpression characteristics and positive correlation of the *PHTF1*, *FEM1b* and *Apaf*-*1* genes in ALL patients and HIs

**Fig. 3:**
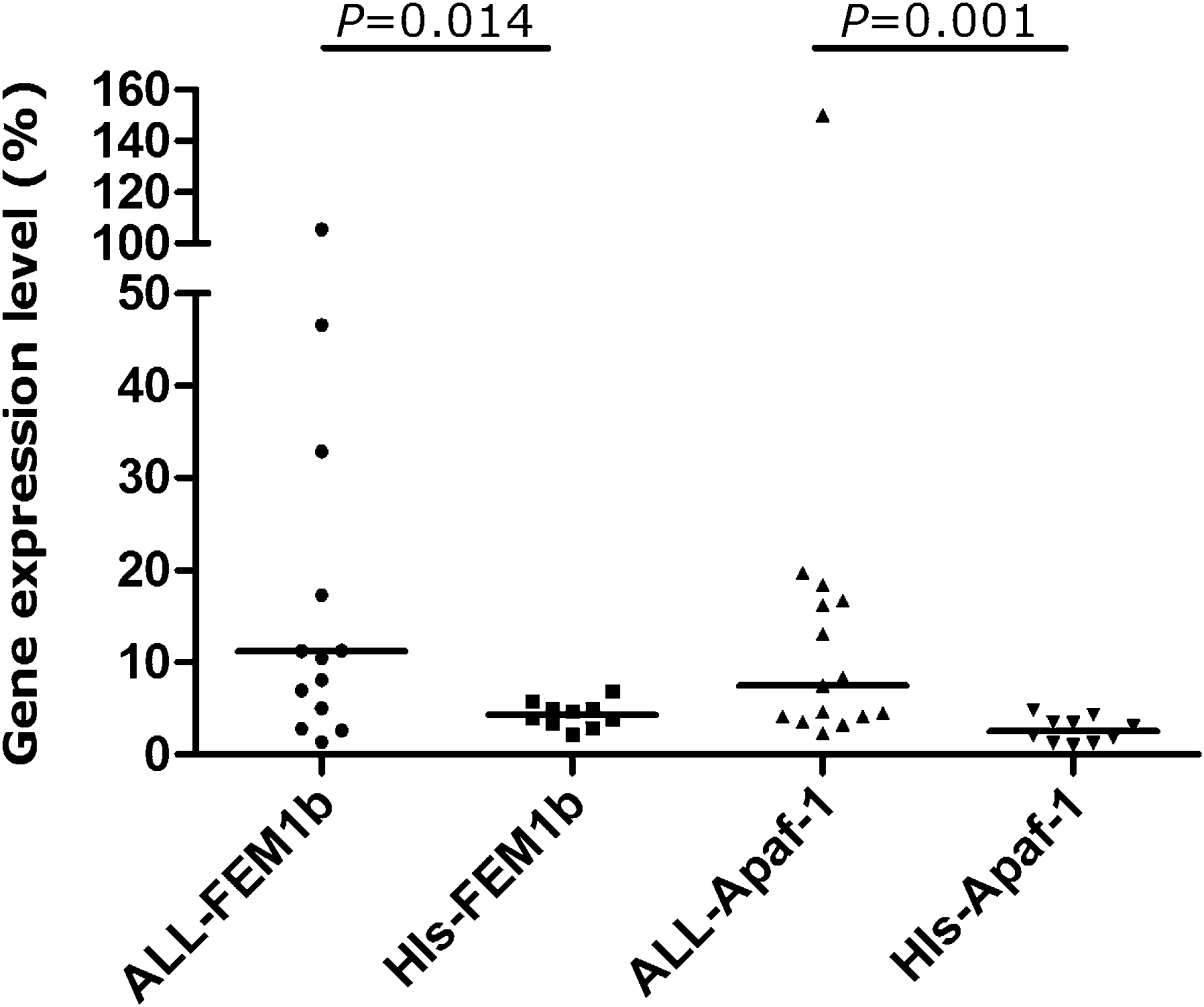
*FEM1b* and *Apaf*-*1* expression levels in the ALL patients and healthy individuals. *FEM1b* and *Apaf*-*1* overexpression was found in recently diagnosed ALL patients versus HIs. *P* = 0.014 for *FEM1b*, *P* = 0.001 for *Apaf*-*1*

*FEM1b* and *Apaf*-*1* overexpression was found in recently diagnosed ALL (*FEM1b* median: 11.19 %, mean rank: 15.90, *P* = 0.014) (*Apaf*-*1* median: 7.47 %, mean rank: 16.87, *P* = 0.001) patients compared with HIs (*FEM1b* median: 4.31 %, mean rank: 8.65) (*Apaf*-*1* median: 2.52 %, mean rank: 7.20) (Fig. 3).

**Fig. 4:**
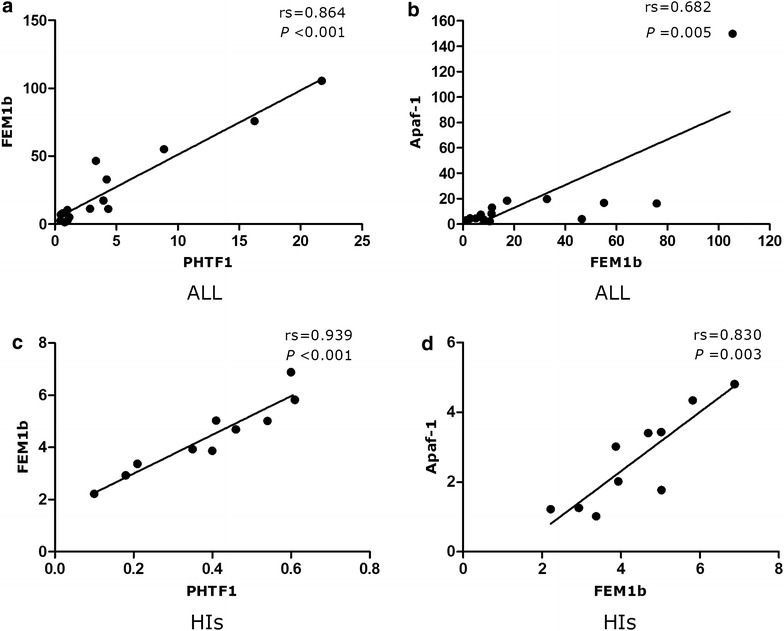
Correlation analysis of the *PHTF1*, *FEM1b* and *Apaf*-*1* expression levels. Positively correlated expression for the *PHTF1*, *FEM1b* and *Apaf*-*1* genes was found in ALL patients (**a** *PHTF1* vs. *FEM1b*, **b** *FEM1b* vs. *Apaf*-*1*) and HIs (**c** *PHTF1* vs. *FEM1b*, **d** *FEM1b* vs. *Apaf*-*1*)

Positively correlated expression for the *PHTF1*, *FEM1b* and *Apaf*-*1* genes was found in ALL patients (*PHTF1* vs. *FEM1b*: rs = 0.864, *P* < 0.001 and *FEM1b* vs. *Apaf*-*1*: rs = 0.682, *P* = 0.005) (Fig. 4a, b) and HIs (*PHTF1* vs. *FEM1b*: rs = 0.939, *P* < 0.001 and *FEM1b* vs. *Apaf*-*1* rs = 0.830 *P* = 0.003) (Fig. 4c, d).

### Inhibition or overexpression of *PHTF1* by lentiviral transduction in T-ALL cell lines

**Fig. 5:**
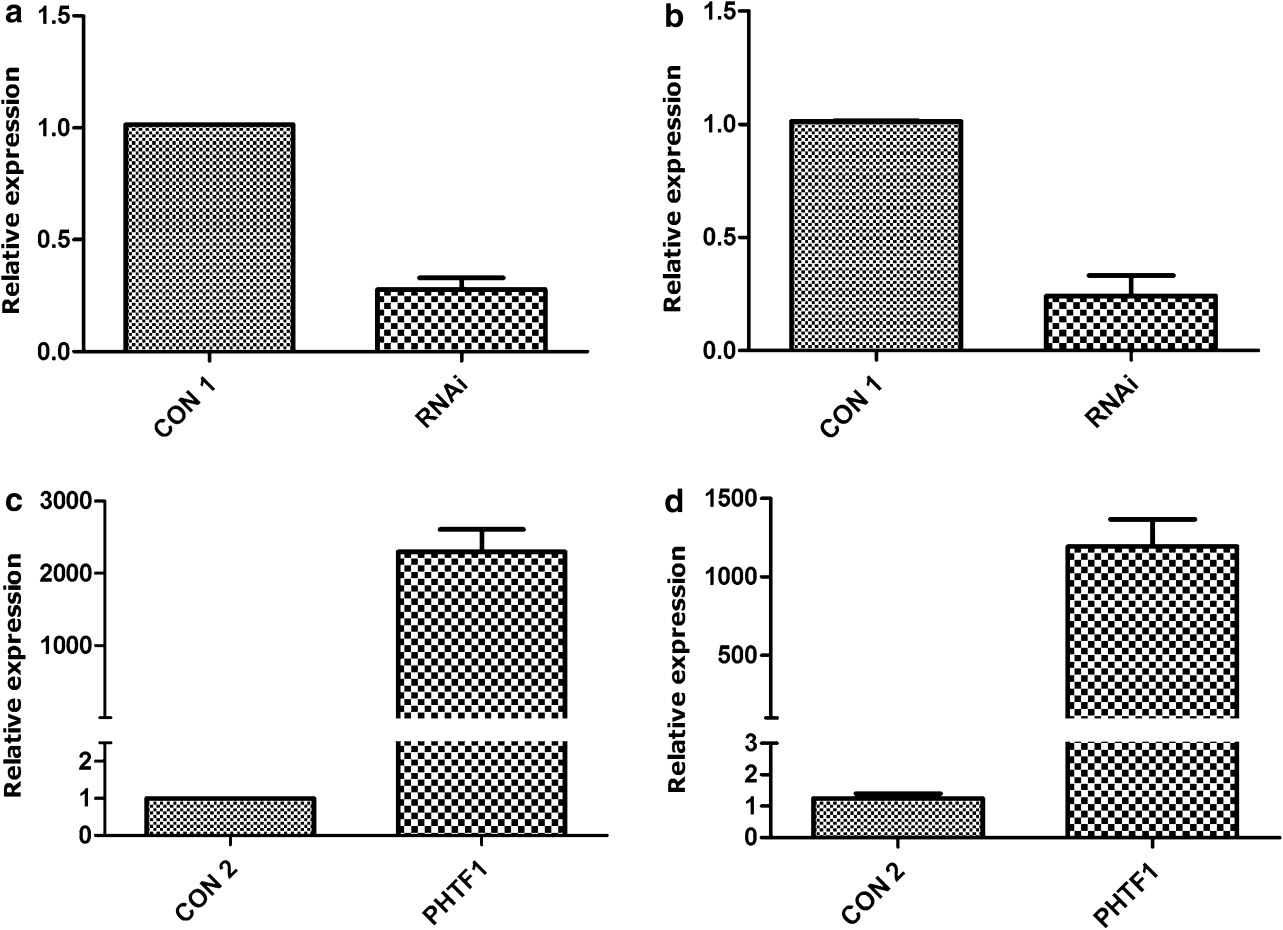
Knockdown and overexpression of PHTF1 in T-ALL cell lines. **a**, **b** *PHTF1* knockdown with *PHTF1* shRNA lentivirus in Jurkat (**a**) and Molt-4 (**b**) cells. *PHTF1* was down-regulated approximately 3-fold in both Jurkat and Molt4 cells. *P* < 0.01 for *bar 1* versus *bar 2* and *bar 3* versus *bar 4*. **c**, **d** *PHTF1* overexpression by *PHTF1* lentivirus in Jurkat (**c**) and Molt-4 (**d**) cells. *PHTF1* was up-regulated approximately 2291-fold in Jurkat cells and approximately 1100-fold in Molt4 cells. *P* < 0.05 for *bar 1* versus *bar 2* and *bar 3* versus *bar 4*. The results represent the mean ± SEM (n = 3)

To investigate the potential role of *PHTF1* in leukemogenesis, we first transfected Jurkat and Molt-4 cells with an active shRNA pair. We found that the *PHTF1* expression level in Jurkat (Fig. 5a) and Molt-4 (Fig. 5b) cells was downregulated approximately 3-fold compared with a scrambled lentiviral transduction control group (CON 1).

Similarly, we transduced Jurkat and molt-4 cells using a lentivirus expressing the *PHTF1* gene. The *PHTF1* expression level was approximately 2291-fold (Jurkat) (Fig. 5c) and approximately 1100-fold (Molt-4) (Fig. 5d) higher compared with the lentiviral transduction control group (CON 2).

### *PHTF1* overexpression inhibits the proliferation of Jurkat and Molt-4 cells

**Fig. 6:**
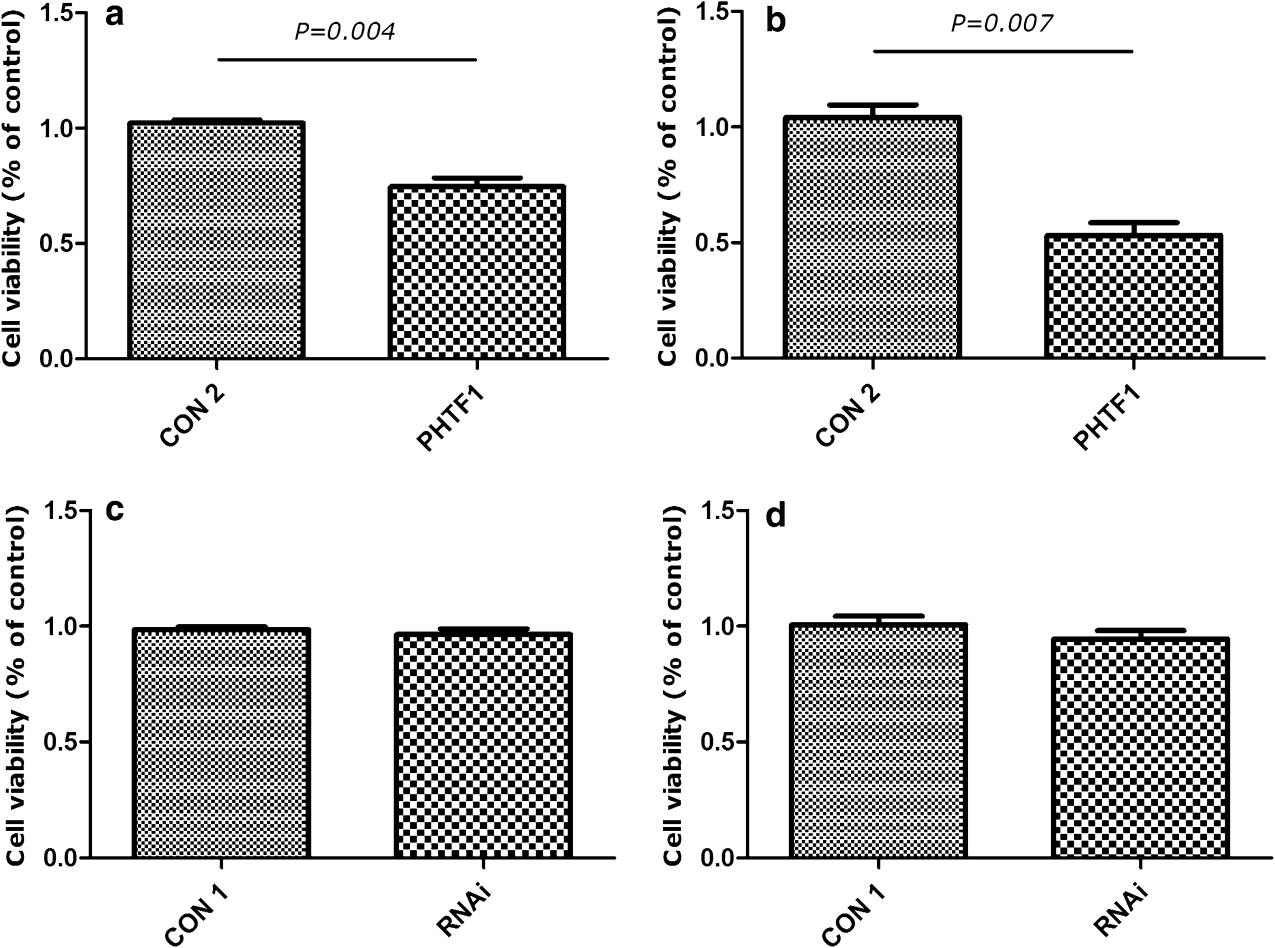
*PHTF1* overexpression inhibits the proliferation of Jurkat and Molt-4 cells. **a** Jurkat cells infected with *PHTF1* (PHTF1) or control (CON 2) lentivirus were seeded in a 96-well plate and incubated for 72 h. The CCK8 assay demonstrated that cells infected with *PHTF1* have lower viability compared with control. The results shown are the mean ± SEM (n = 3). *P* = 0.004 for *bar 1* versus *bar 2*. **b** Molt-4 cells infected with *PHTF1* (PHTF1) or control (CON 2) lentivirus were seeded in a 96-well plate and incubated for 72 h. The CCK8 assay demonstrated that cells infected with *PHTF1* have lower viability compared with controls. The results represent the mean ± SEM (n = 3). *P* = 0.007 for *bar 1* versus *bar 2*. **c** Jurkat cells infected with *PHTF1* shRNA (RNAi) or control (CON 1) lentivirus were seeded in a 96-well plate and incubated for 72 h. The CCK8 assay demonstrated that cells infected with *PHTF1* shRNA have no significant difference compared with controls. The results represent the mean ± SEM (n = 3). *NS* no significance. **d** Molt4 cells infected with *PHTF1* shRNA (RNAi) or control (CON 1) lentivirus were seeded in a 96-well plate and incubated for 72 h. The CCK8 assay demonstrated that cells infected with *PHTF1*-shRNA have no significant difference compared with controls. The results represent the mean ± SEM (n = 3). *NS* no significance

The effect of *PHTF1* on Jurkat and Molt-4 cell growth was next evaluated. Compared with the negative control group, *PHTF1* had markedly lower proliferation in both Jurkat (n = 3, *P* = 0.004) (Fig. 6a) and Molt-4 cells (n = 3, *P* = 0.007) (Fig. 6b). However, we found no obvious proliferation inhibition after *PHTF1* knockdown in Jurkat (Fig. 6c) and Molt-4 cells (Fig. 6d).

### *PHTF1* overexpression remarkably induces apoptosis in Molt-4 cells

**Fig. 7:**
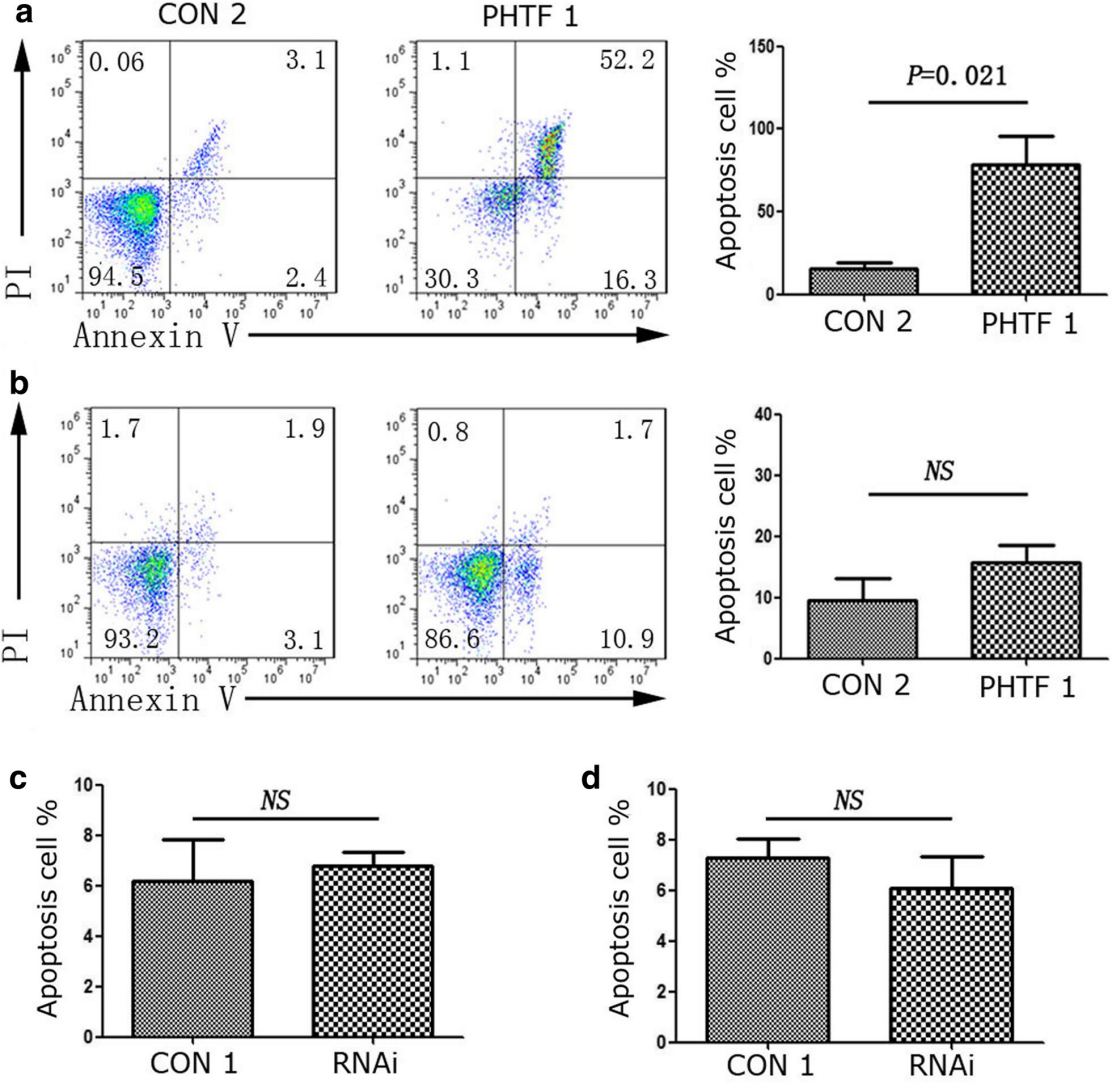
*PHTF1* overexpression induces apoptosis in Molt-4 cells. **a** Representative FACS plots and summary (*right*) of apoptotic Molt 4 cells after transduction with *PHTF1* (PHTF1, *left*) or control (CON 2, *middle*) lentivirus. Data represent the mean ± SEM (n = 3). P = 0.021 for *bar 1* versus *bar 2*. **b** Representative FACS plots and summary (*right*) of apoptotic Jurkat cells after transduction with *PHTF1* (PHTF1, *left*) or control (CON 2, *middle*) lentivirus. Data represent the mean ± SEM (n = 3). *NS* no significance. **c**, **d** Summary of apoptotic Jurkat (**c**) or Molt-4 (**d**) cells after transduction with *PHTF1* shRNA (RNAi) or control (CON 1) lentivirus. Data represent the mean ± SEM (n = 3). *NS* no significance

We then explored the effects of *PHTF1* overexpression on T-ALL apoptosis in in vitro assays. The ratio of apoptosis in Molt-4 cells was 78.67 ± 29.26 %, and it was 15.50 ± 5.97 % for the negative control group (n = 3, *P* = 0.021) (Fig. 7a). Interestingly, the apoptosis ratio for Jurkat cells was 15.87 ± 5.65 %, while it was 9.57 ± 2.35 % for the negative control group. No significant difference between these two groups was found (n = 3, *P* = 0.150) (Fig. 7b). Notably, we could observe an increase in the trend toward apoptosis for Jurkat cells after *PHTF1* upregulation; however, we found no significant apoptosis after *PHTF1* knockdown in the Jurkat (Fig. 7c) and Molt-4 cells (Fig. 7d).

### *PHTF1*-related gene expression in Jurkat and Molt-4 overexpressing cells

**Fig. 8:**
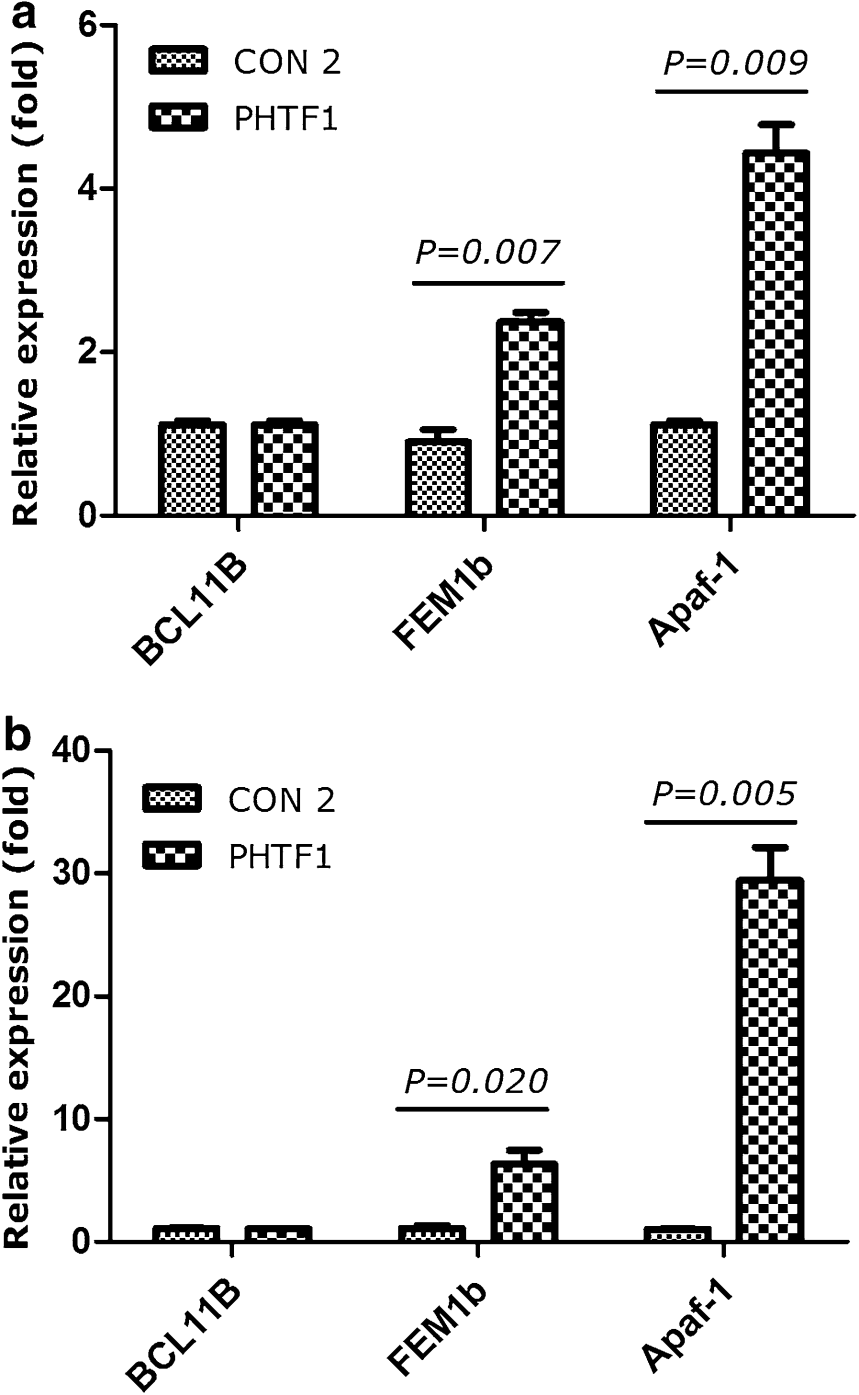
Genes activated with *PHTF1* overexpression. **a** Expression level of *BCL11B*, *FEM1b* and *Apaf*-*1* in Jurkat cells after *PHTF1* overexpression by transduction with *PHTF1* lentivirus (PHTF1) or in the presence of control lentivirus (CON 2). The *BCL11B* expression level was unchanged versus control; *FEM1b* and *Apaf*-*1* were up-regulated 2.4- and 4.4-fold, respectively, versus control. Data shown are the mean ± SEM (n = 3), *P* = 0.007 for *bar 3* versus *bar 4*; *P* = 0.009 for *bar 5* versus *bar 6*. **b** Expression levels of *BCL11B*, *FEM1b* and *Apaf*-*1* in Molt-4 cells after *PHTF1* overexpression by PHTF1 lentivirus (PHTF1) or control lentivirus (CON 2). The *BCL11B* expression level was unchanged versus control; *FEM1b* and *Apaf*-*1* were up-regulated 6.3- and 29.4-fold, respectively, versus control. Data shown are the mean ± SEM (n = 3), *P* = 0.020 for *bar 3* versus *bar 4*; *P* = 0.005 for *bar 5* versus *bar 6*

To further examine the mechanism by which *PHTF1* regulates apoptosis, we investigated the expression of *PHTF1*-related genes in *PHTF1* (PHTF1) or control (CON 2) infected Jurkat and Molt-4 cells. In infected Jurkat cells, the *BCL11B* expression level was unchanged, while *FEM1b* and *Apaf*-*1* were upregulated approximately 2.4- and 4.4-fold, respectively (Fig. 8a). In transfected Molt-4 cells, the *BCL11B* expression level was also unchanged, while *FEM1b* and *Apaf*-*1* were upregulated approximately 6.3- and 29.4-fold, respectively (Fig. 8b).

## Discussion

Despite significant improvement in our understanding of T-ALL biology and pathogenesis [25], knowledge of the T cell activative signaling pathways involved in T-ALL remains limited. Thus, novel molecular insights and therapeutic approaches are urgently needed. Thus, based on previous finding that *BCL11B* siRNA-mediated apoptosis in the Molt-4 T-ALL cell line might be related to the *PHTF1* gene [10], we focused our attention on the characteristics of *BCL11B* and *PHTF1* gene expression in T-ALL patients. We found a positive correlation between the *PHTF1* and *BCL11B* genes in healthy individuals. In contrast, a trend toward a negative correlation was found for the *PHTF1* and *BCL11B* genes in the *T*-*ALL* patient although there was no statistical significance, which may be due to the limited number of samples. However, this result may indicate that there are different expression patterns for both of these genes in T-ALL, and for further confirmation of the relationship between *PHTF1* and *BCL11B* is needed in a larger cohort of T-ALL samples. Low *BCL11B* expression is associated with poor prognosis, particularly in the standard risk group for thymic T-ALL [26]. *PHTF1* and *BCL11B* genetic disorders may contribute to T-ALL pathogenesis.

To further explore its function in T-ALL cell lines, we downregulated and overexpressed *PHTF1* in T-ALL cell lines and examined cell line growth and apoptosis using in vitro assays. *PHTF1* up-regulation inhibits the proliferation of Jurkat and Molt-4 cells and effectively induces apoptosis in Molt-4 cells. The *BCL11B* expression level was unchanged, while *FEM1b* and *Apaf*-*1* were upregulated. Interestingly, compared with the Jurkat cells, the remarkable apoptosis of Molt-4 cells may be related to the higher *FEM1b* and *Apaf*-*1* expression level. Based on the reports regarding the expression characteristics of tumor suppressor genes in leukemia, such as wilms tumor 1 (*WT1*), which was consistently found to be highly expressed in peripheral blood (PB) or bone marrow (BM) in acute myeloid leukemia (AML) and is used for inhibiting tumor targeting [27], we hypothesized that *PHTF1* is involved in negative regulation of tumor growth. Therefore, we considered that *PHTF1* has tumor-suppressive activity and triggers the *PHTF1*-*FEM1b*-*Apaf*-*1* apoptosis pathway using in vitro assays. However, direct inhibition of *PHTF1* expression, and the *BCL11B* expression level was unchanged (data not shown), had no significant effect on the proliferation or apoptosis of Jurkat and Molt-4 cells. Therefore, we considered that *PHTF1* might be the downstream gene of the *BCL11B*.

In order to further characterize the role of PHTF1 in T-ALL, it is of interest to analyze the downstream genes regulated by *PHTF1*. Because overexpression of *PHTF1* was found in both groups (T-ALL and B-ALL) in comparison with HIs group, we characterized the expression of *FEM1b* and *Apaf*-*1*, which are mainly involved in apoptosis in ALL patients and HIs. In a previous study [22], western blotting and immunofluorescence assays revealed the presence of *PHTF1* and *FEM1b* in the same cells, and association between these proteins was demonstrated by co-immunoprecipitation. Previous in vitro experiments have suggested that the human *FEM1b* gene is involved in apoptosis. Human *FEM1b* is 99 % identical to the mouse protein, and it is reportedly capable of associating with the intracellular tail of the death membrane receptors *Tnfrsf6* (tumor necrosis factor receptor superfamily, member 6; also known as Fas) and *Tnfrsf1a* (also known as TNFR1) [28]. Proteasome inhibitor treatment of SW620, HCT-116, and DLD-1 cells led to upregulation of the *FEM1b* protein and was associated with apoptosis induction. Blockade of *FEM1b* upregulation with morpholino antisense oligonucleotides suppressed proteasome inhibitor-induced apoptosis in these cells. The authors of this study have demonstrated that *FEM1b* can induce apoptosis when overexpressed in some cell lines. The proapoptotic protein *FEM1b* could represent a novel molecular target for overcoming apoptosis resistance in colon cancer therapy [29]. As a binding protein for *FEM1b*, *Apaf*-*1* is a central component of the intrinsic apoptosis pathway. High *Apaf*-*1* expression elevates erythroid apoptosis in iron overload myelodysplastic syndrome [30]. Zermati et al. have suggested that *Apaf*-*1* deficiency contributes to tumor progression not only by decreasing activation of the apoptotic caspase but also by reducing DNA damage-induced cell cycle arrest, thus weakening the cytostatic effects of chemotherapy and radiotherapy [31]. *FEM1b* and *Apaf*-*1* overexpression was found in samples from patients recently diagnosed with ALL, and a positively correlated expression level for the *PHTF1*, *FEM1b* and *Apaf*-*1* genes was found in ALL patients and HIs. Thus, *PHTF1*, *FEM1b*, and *Apaf*-*1* might be involved in the same apoptosis pathway. The data provide the first molecular biological characterization of these genes at the gene expression level. To further explore its function in B-ALL,further studies will be performed to address this question.

## Conclusions

In conclusion, *PHTF1* overexpression is responsible for regulating the cell proliferation and apoptosis of T-ALL cell lines. Based on these preliminary findings, and reports regarding the expression characteristics of tumor suppressor genes in leukemia, such as wilms tumor 1 (*WT1*), which was consistently found to be highly expressed in peripheral blood (PB) or bone marrow (BM) in acute myeloid leukemia (AML) and is used for inhibiting tumor targeting, we hypothesized that *PHTF1* is involved in negative regulation of tumor growth. *PHTF1* may be a tumor-suppressor like gene and a therapeutic target for triggering the *PHTF1*-*FEM1b*-*Apaf*-*1* apoptosis pathway.

## Methods

### Samples

Nine newly diagnosed and untreated patients with T-ALL, and thirteen newly diagnosed and untreated patients with B-ALL were recruited. The diagnoses for all patients were based on cytomorphology, immunohistochemistry, cytoimmunological and cytogenetic analysis. Peripheral blood mononuclear cells (PBMCs) from ten healthy individuals (HIs) served as controls. The details of the samples are listed in Table 1. Peripheral blood was collected by heparin anticoagulation, and PBMCs were separated using the Ficoll-Hypaque gradient centrifugation method. All procedures were conducted in accordance with the guidelines of the Medical Ethics committees of the Health Bureau of Guangdong Province, China.

**Table 1:** The details of samples used in study

Diagnosis	Subtype	Numbers			Age (year)	
		Total	Male	Female	Range	Median
ALL		22	17	5	15–55	22.5
	T-ALL	9	7	2	17–27	20
	B-ALL	13	10	3	15–55	24
HIs		10	6	4	22–35	25.5

### Cell culture

Cells from the well-characterized human T-cell acute lymphoblastic leukemia (T-ALL) cell lines Jurkat and Molt-4 were obtained from American Type Culture Collection (ATCC, Manassas, Virginia, USA) and maintained in RPMI-1640 media (Gibco, New York, USA) containing 10 % fetal bovine serum (FBS, NATOCOR, Argentina) at 37 °C and 5 % CO_2_.

### Lentivirus production and transduction

Two recombinant lentivirus vectors based on the U6-shRNA-Ubi-EGFP and U6-NC-Ubi-EGFP vectors (purchased from Shanghai GeneChem Co., Ltd) were constructed to target the human *PHTF1* gene (NM_006608) and as a scrambled negative control, respectively. The target sequence of the *PHTF1* shRNA was *ACCTAAACTCTCAGGTAAA*, and that for the negative control was *TTCTCCGAACGTGTCACGT.*

Two recombinant lentivirus vectors based on the Ubi-PHTF1-3FLAG-SV40-EGFP and Ubi-3FLAG-SV40-EGFP vectors (purchased from Shanghai GeneChem Co., Ltd) were constructed to express the human *PHTF1* gene (NM_006608) and green fluorescence protein gene (negative control), respectively.

The transduced cells were divided into four groups: shRNA transduction (RNAi), scrambled control (CON 1), *PHTF1* gene transduction (PHTF1), and transduction control (CON 2). The lentiviral titers ranged from 1 to 2 × 10^8^ TU/mL. Transduction was performed by ‘spin-infection’, and cells and lentiviruses (MOI = 5 for Jurkat, MOI = 10 for molt4) plus 10 μg/mL polybrene were mixed and spun at 900 × g for 40 min. The transfected cells were then cultured for 2–3 days prior to analysis by flow cytometry. For GFP cell analysis, GFP+ cells were cultured for 3 days and then FACS purified using the Aria II System (Becton–Dickinson Immunocytometry Systems, San Jose, CA, USA).

### RNA extraction and cDNA synthesis

RNA was extracted using the TRIzol kit (Invitrogen, Carlsbad, CA, USA), and it was then reverse-transcribed into first-strand cDNA using random hexamer primers and the reverse transcriptase Superscript II Kit (TaKaRa, Dalian, PR China) according to the manufacturer’s instructions.

### Real-time polymerase chain reaction

Real-time quantitative reverse transcription-polymerase chain reaction (qRT-PCR) quantitative detection of the β2 microglobulin (*β2M)*, *BCL11B*, *PHTF1*, *FEM1b*, and *Apaf*-*1* genes in cDNA from PBMCs was performed using TaqMan real-time PCR. To precisely determine the *BCL11B* copy number, a duplex vector, including a fragment of the *BCL11B* and *β2M* genes was constructed and used as a reference (the duplex vector was a gift from Prof. C. A. Schmidt, Ernst-Moritz-Arndt University Greifswald, Germany). Another vector including the *PHTF1*, *FEM1b*, and *Apaf*-*1* genes was constructed based on DNA concentration, and it was measured by spectrophotometry and confirmed by quantitative gel electrophoresis. Standard dilutions of the vector ranging from 10^7^ to 10^1^ copies were prepared.

Briefly, PCR was performed in a 25 μL total volume containing 2 μL cDNA, 25 pmol of each primer, 10 nmol of each dNTP, 1.5 U AmpliTaq Gold (Applied Biosystems, Branchburg, NJ, USA), 5 pmol 6FAM-TAMRA probe, and PCR buffer containing 4.5 mM MgCl_2_. After an initial denaturation at 95 °C for 5 min, 50 cycles of 95 °C for 15 s and 64 °C for 1 min were performed.

Primers and probes for *β2M*, *BCL11B*, *PHTF1*, *FEM1b*, and *Apaf*-*1* gene amplification were synthesized by Invitrogen (Carlsbad, CA, USA) (Table 2). The absolute amounts of *BCL11B* and *β2M* were measured in two independent assays, and the *BCL11B* content per 100,000 *β2M* copies was calculated using the following formula: n = 100,000 × *BCL11B*/*β2M*. The amounts of the *PHTF1*, *FEM1b*, and *Apaf*-*1* copies were calculated using the following formulas: n = *PHTF1*/*β2M* × 100 %, n = *FEM1b*/*β2M* × 100 %, and n = *Apaf*-*1*/*β2M* × 100 %.

**Table 2:** Sequences of primers and probes for real-time PCR (TaqMan method)

Primer/probe	Sequence
BCL11B-F	5′-CACCCCCGACGAAGATGACCAC-3′
BCL11B-R	5′-CGGCCCGGGCTCCAGGTAGATG-3′
BCL11B-P	5′-FAM-TCACCCACGAAAGGCATCTGTCCCAAGCA-TAMRA-3′
β2M-F	5′-CTCGCGCTACTCTCTCTTTCT-3′
β2M-R	5′-TACATGTCTCGATCCCACTTAACTAT-3′
β2M-P	5′-FAM-CTCACGTCATCCAGCAGAGAATGGAAAGTCA-TAMRA-3′
PHTF1-F	5′-GGAAAGTGATGACTGCAGAAACC-3′
PHTF1-R	5′-AACACCATTCATTCGCTTTGG-3′
PHTF1-P	5′-FAM-CCTGCTTGTTCACATGCACAGGTGC-TAMRA-3′
FEM1B-F	5′-CACTCCATCATCATTAGCCTAGTTGA-3′
FEM1B-R	5′-TGTACTTTTGTCTAGCGGAGTCTTATTCT-3′
FEM1B-P	5-FAM-CCGGAGCTCACACTGACATGACGAATAA-TAMRA-3′
APAF1-F	5′-TGCGCTGCTCTGCCTTCT-3′
APAF1-R	5′-CATGGGTAGCAGCTCCTTCTTC-3′
APAF1-P	5′-FAM-TGAGCTTCTTCATTTGTGTGCTCCGCT-TAMRA-3′

The absolute amounts of *BCL11B* and *β2M* were measured in two independent assays, and the *BCL11B* content per 100,000 *β2M* copies was calculated using the following formula: n = 100,000 × *BCL11B/β2M* [12]. In this study, we used this method to measure *BCL11B* expression. The standard for other genes (*PHTF1*, *FEM1B* and *Apaf*-*1*) was constructed using another triplex plasmid (synthesized by Invitrogen), and we determined the *β2M*, *PHTF1*, *FEM1B* and *Apaf*-*1* copy number, which were compared relative to the gene expression level of the *β2M* reference gene between different clinical samples.

### Cell proliferation assay

Cell proliferation was detected using the Cell Counting Kit-8 (CCK-8) assay according to the manufacturer’s instructions. Briefly, Jurkat and Molt-4 cells transfected with or without lentiviruses at a density of 5 × 10^5^ cells/mL were seeded into a 96-well plate (100 µL/well). CCK-8 reagent (10 µL) was then added to each well, and the cells were cultured for 3 h at 37 °C and 5 % CO_2_. After incubation, the absorption value (450 nm) of each well was measured with a 680-type microplate reader (Bio-Rad, Berkeley, CA, USA).

### Apoptosis assays

Annexin-V binding assays were conducted 72 h after lentiviral transduction using the Apoptosis Detection Kit (eBioscience, San Diego, CA, USA) according to the manufacturer’s instructions. Briefly, cells were washed twice with PBS, resuspended in 100 μL binding buffer containing APC-conjugated Annexin V, and incubated in the dark for 15 min. Cells were then washed and suspended in 200 μL binding buffer containing PI. Annexin V positive cells were analyzed with an Accuri C6 flow cytometer (Becton–Dickinson Immunocytometry Systems, San Jose, CA, USA).

### Statistical analyses

Differences in mRNA expression between two clinical groups were analyzed by the Mann–Whitney U test. Data are presented as medians. Spearman’s rank correlation analysis was used to analyze the *PHTF1*, *BCL11B*, *FEM1b*, and *Apaf*-*1* mRNA level in different clinical samples. An independent-sample t test was used for all comparisons, and data are represented as mean ± SD. *P* values <0.05 were considered statistically significant.

## Abbreviations

ALL: acute lymphoblastic leukemia
Apaf-1: apoptotic protease activating factor-1
ATCC: American Type Culture Collection
B-ALL: B-cell acute lymphoblastic leukemia
β2M: β2 microglobulin
BCL11B: B-cell leukemia/lymphoma 11B
CCK8: Cell Counting Kit-8
FEM1b: feminization-1 homolog b
HIs: healthy individuals
PBMCs: peripheral blood mononuclear cells
PHTF1: putative homeodomain transcriptional factor
qRT-PCR: real-time quantitative reverse transcription-polymerase chain reaction
siRNA: small interference RNA
shRNA: small hairpin RNA
T-ALL: T-cell acute lymphoblastic leukemia
TNFR1: tumor necrosis factor receptor-1
